# A Sensitive LC–MS/MS Method for Quantifying Beta‐Casomorphin 5 in Pasteurized Milk

**DOI:** 10.1002/fsn3.72484

**Published:** 2026-10-19

**Authors:** Duc Doan Nguyen, Vicky Solah, Suzanne Daubney, Malgorzata Kowalczyk, Wendy Hunt

**Affiliations:** ^1^ Food Science and Nutrition, School of Medical, Molecular and Forensic Sciences Murdoch University Murdoch Western Australia Australia; ^2^ Centre for Crop and Food Innovation, Food Futures Institute Murdoch University Murdoch Western Australia Australia; ^3^ Bannister Downs Dairy Northcliffe Western Australia Australia; ^4^ School of Maths, Statistics, Chemistry and Physics Murdoch University Murdoch Western Australia Australia

**Keywords:** beta‐casomorphin 5, heat treatment, LC–MS/MS, pasteurized milk, solid‐phase extraction

## Abstract

This study aimed to apply a highly sensitive liquid chromatography–tandem mass spectrometry (LC–MS/MS) method to quantify beta‐casomorphin 5 (β‐CM5) content in pasteurized milk (PM) and to evaluate the effect of heat treatment on this peptide. Commercial PM samples were collected from local supermarkets, and heat treatment of industrial milk (IP) samples was performed at 62°C and 72°C for 5 min. The milk samples were purified using solid‐phase extraction (SPE) and analyzed for β‐CM5 using the LC–MS/MS combined with stable isotope‐labeled internal standards. The method achieved high sensitivity, with a limit of detection of 0.03 ng/g. The concentration of β‐CM5 ranged from 0.10 to 0.55 ng/g for commercial PM samples and from 0.10 to 0.13 ng/g for IP samples. These results suggest that industrial heat treatment had a very slight effect on β‐CM5, and variation in this peptide content in PM samples is primarily driven by differences in raw milk origin, such as endogenous enzyme activity and beta‐casein content, rather than pasteurization conditions.

## Graphical abstract

Beta‐casomorphin 5 (β‐CM5) in pasteurized milk was quantified using sensitive LC–MS/MS. The findings provide new insights into β‐CM5 occurrence in pasteurized milk and suggest that pasteurization treatments were unlikely to promote β‐CM5 release.

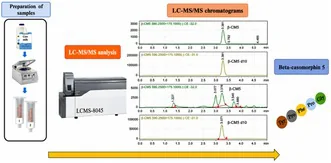

## 1 Introduction

Beta‐casomorphin 5 (β‐CM5) is an opioid pentapeptide (Figure 1), which is released from bovine beta‐caseins (β‐caseins) during the processing of milk, in particular cheese‐making (Sienkiewicz‐Szłapka et al. 2009). In the intestine, this pentapeptide is also released from beta‐casomorphin 7 (β‐CM7) by the dipeptidyl peptidase‐IV (DPP‐IV) enzyme (Shivam et al. 2024). β‐CM5 exhibits a higher affinity, compared to β‐CM7, for μ‐opioid receptors (Brantl et al. 1979, 1981). Several studies have reported the potential adverse impacts of β‐CM7 on human health (Elliott et al. 1999; Laugesen and Elliott 2003; Sokolov et al. 2014; Pal et al. 2015; Jianqin et al. 2016), while few studies have investigated the relationship between β‐CM5 and human health. An epidemiological study suggests that β‐CM5 may be associated with apnoea in infants (Wasilewska et al. 2011). However, the data obtained from in vitro and ex vivo studies on the human health impacts of beta‐casomorphin (β‐CM), especially β‐CM5 and β‐CM7, remain inconclusive and insufficient to fully support their potential adverse effects on human health (Daniloski et al. 2021; Simonetta et al. 2025).

**FIGURE 1:**
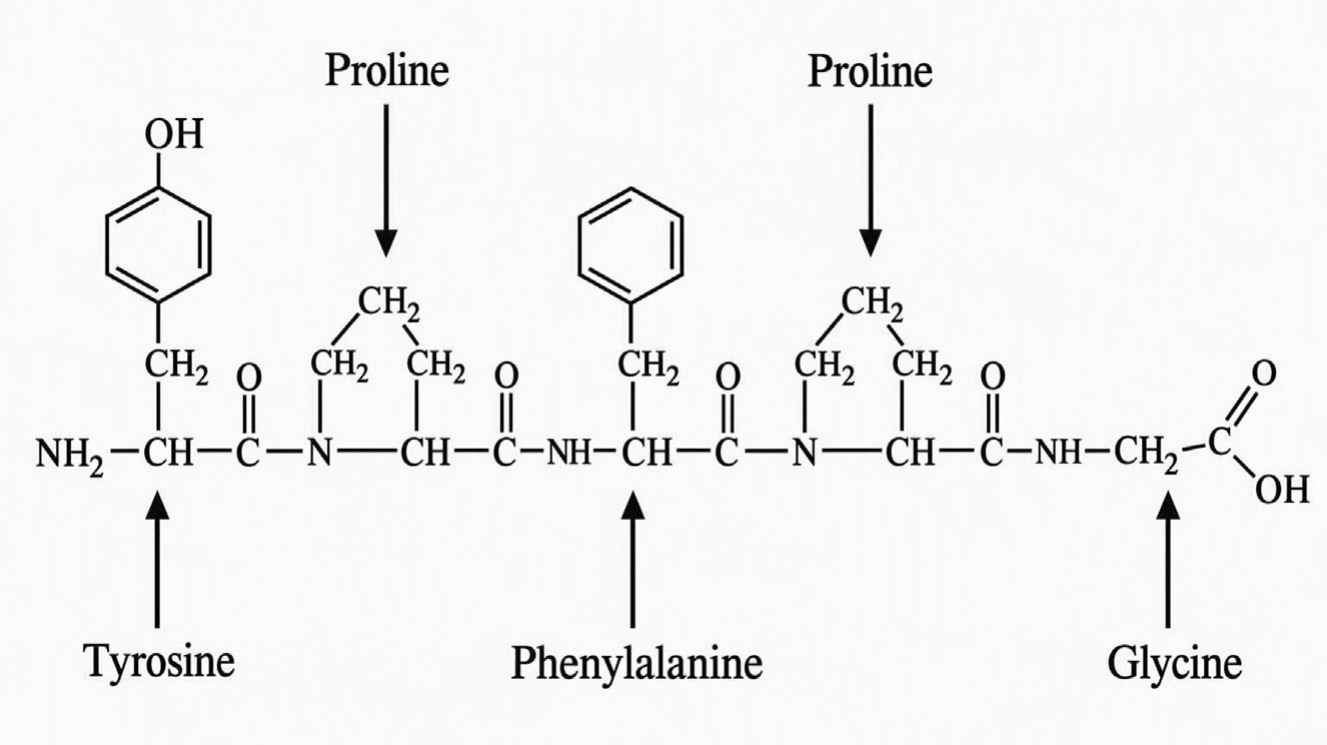
Chemical structure of β‐casomorphin 5 (Adapted from Juan‐García et al. 2009). The peptide is derived from β‐casein, consisting of the amino acid sequence Tyr‐Pro‐Phe‐Pro‐Gly. The positions of key amino acid residues, including tyrosine, proline, phenylalanine, and glycine, are indicated.

The presence of β‐CM5 in various dairy products remains controversial and depends on the sensitivity of the analytical method used. There is a range of analytical techniques being used to detect β‐CM5 in dairy products and gastrointestinal digests of dairy products (Juan‐García et al. 2009; Sienkiewicz‐Szłapka et al. 2009; De Noni and Cattaneo 2010; De Noni et al. 2015; Nguyen et al. 2015, 2021; Lambers et al. 2021; Simonetta et al. 2025). Sienkiewicz‐Szłapka et al. (2009) applied an enzyme‐linked immunosorbent assay (ELISA) for the quantification of β‐CM5 in cheeses such as Brie, Rokpol, Edamski, Gouda, and Kasztelan with contents ranging from 0.05 to 3.14 μg/g of cheese. In contrast, using liquid chromatography–tandem mass spectrometry (LC–MS/MS), De Noni and Cattaneo (2010) did not detect this pentapeptide in various cheeses such as Gorgonzola, Caprino, Brie, Gouda, Taleggio, Fontina, Cheddar, and Grana Padano. However, β‐CM5 has been recently found in Gorgonzola cheese (De Noni et al. 2015) or in Bergader and Stilton cheese (Simonetta et al. 2025).

Analysis of liquid milk has also produced different results for β‐CM5. According to Thiruvengadam et al. (2021), β‐CM5 content in raw milk is highest in colostrum and lower in mature milk. Using LC‐MS/MS, Nguyen et al. (2019) quantified β‐CM5 content in the range of 0.0 and 0.64 ng/g in raw milk. However, this peptide was not detected in pasteurised and ultra‐high temperature milk (UHT) using either LC‐MS/MS or high‐resolution liquid chromatography‐mass spectrometry (HRLC‐MS) (Juan‐García et al. 2009; De Noni and Cattaneo 2010; 2015; Nguyen et al. 2015). The variation in β‐CM5 content observed in published studies may result from milk processing conditions (milk origin, bacteria and aging of cheese) or analytical methods used for determining β‐CM5. To the best of our knowledge, no study has reported the presence of β‐CM5 in pasteurised milk and the influence of commercial heat treatment on this peptide content.

The present study aimed to apply a highly sensitive method to identify and quantify β‐CM5 in pasteurized milk and to examine variations in β‐CM5 concentrations following industrial heat treatments. Initially, an LC–MS/MS method was developed using an isotope‐deuterated compound as an internal standard for the identification/quantification of β‐CM5. This optimized method included a highly purified sample preparation step involving solid‐phase extraction (SPE) followed by analysis using LC–MS/MS.

## 2 Materials and Methods

### 2.1 Chemicals and Materials

Commercial full‐fat pasteurized milk (PM) samples were collected from local supermarkets in Western Australia (WA). These dairy products were processed by the five dairy processing plants in different regions in WA. All PM samples were transported to the laboratory and then fifty grams of each were immediately frozen at −20°C overnight before freeze‐drying. The samples of freeze‐dried powder were weighed accurately and stored at −20°C until analyzed (Table S1). Raw milk and heated milk samples, called industrial milk (IP) samples which were industrially processed at 62°C/5 min and 72°C/5 min, were provided by a dairy processor. All IP samples packed in airtight plastic bags were transported in an ice box to the laboratory in 6 h. At the laboratory, IP samples were immediately processed for the extraction of β‐CM5. The β‐CM5 standard and stable isotope labeled [^2^H10] β‐CM5 (β‐CM5‐d_10_) standards (Bachem, Bubendorf, Switzerland) used as the internal standard were purchased from Auspep Pty (Tullamarine, Vic, Australia). Methanol (MeOH) (RCI LabScan, Bangkok, Thailand) and ultra‐pure water (Honeywell, USA) were LC–MS grade and purchased from ChemSupply (Grillman, SA, Australia).

### 2.2 Extraction of Peptides

Beta‐casomorphin 5 was extracted as described by Nguyen et al. (2019) with some modifications. The milk powder derived from PM samples (approximately 1 ± 0.0001 g) shown in Table S1 was reconstituted using 10 mL of Milli‐Q water. The reconstituted milk, IP samples (approximately 10 ± 0.0001 g) were spiked with β‐CM5‐d_10_ standard (500 ng) and thoroughly mixed using a vortex. All spiked milk samples were adjusted for pH to 4.5–4.6 using 1 mol/L HCl and then left for 10 min at room temperature to completely precipitate protein caseins. The samples were centrifuged at 10,000 × g for 10 min at 4°C. The supernatants were decanted and filtered through Whatman filter papers (N^o^ 1). The filtrates were made up to a final volume of 20 mL with Milli‐Q water. The diluted filtrates were thoroughly mixed using a vortex and then loaded onto the SPE cartridges (Strata‐X‐PRO, 500 mg/6 mL, Phenomenex, NSW, Australia). Interfering compounds were washed away from the SPE cartridges using 4.5 mL of 10% MeOH in Milli‐Q water (v/v). After being eluted using 11.5 mL of MeOH, the eluents were evaporated at 30°C in CentriVap coupled with ColdTrap (Labconco, Kansas, US) until dry. The residues were reconstituted with 1 mL of Milli‐Q water/MeOH mixture (70:30; v/v) in an ultrasonic bath at 30°C for 30 min. The extracts were filtered through 0.22 μm PTFE hydrophilic membrane filters (Velocity Scientific Solutions, Vic, Australia) before LC–MS/MS analysis. Analysis of each milk sample was performed in duplicate.

### 2.3 Liquid Chromatography–Tandem Mass Spectrometry Analysis

Beta‐casomorphin 5 content was analyzed using the UHPLC Nexera X2 system coupled with a Triple quadrupole mass spectrometer (LCMS‐8045, Shimadzu, Japan). The specific configuration includes two LC‐30 ad pumps, two DGU‐20A5R online degassing units, SIL‐30 AC autosampler, CTO‐30A column oven, CMB‐20A system controller, and LCMS‐8045 mass spectrometer equipped with an electrospray ionization interface (ESI).

The chromatographic separation for β‐CM5 was conducted using an ACQUITY UPLC BEH C18 column (100 mm × 2.1 mm, 1.7 μm particle size) at 35°C. The injection volume was 5 μL. Ultra‐pure water (A) and MeOH (B), both containing 0.1% formic acid, were used as the mobile phase. The flow rate was 0.4 mL/min. The gradient was set as follows: 0–0.5 min 30% B; 0.5–5 min 75% B; 5–6 min 75% B; 6–8 min 100% B; 8–8.20 min 30% B; 8.20–12 min 30% B.

Data acquisition and quantitative analysis were carried out using LabSolution version 5.113 software (Shimadzu, Japan). Data were collected using multiple reaction monitoring (MRM) analysis in positive ionization mode (ESI+) under the following conditions: nebulizer gas flow, drying gas flow, and heating gas flow were 2, 10, 10 L/min, respectively. DL temperature, interface temperature, and heat block temperature were 230°C, 300°C, and 400°C, respectively. CID gas and interface voltage were 230 kPa and 4 kV, respectively. The precursor ion, product ion, pre‐bias voltages (Q1 and Q3), and collision energy (CE) were optimized for β‐CM5 and β‐CM5‐d_10_ using an automated MRM optimization program with LabSolution workstation. One multi‐component method has been created for simultaneous analysis. The most abundant MRM transition was used for quantification (Q), and the second transition was used for identification (q). The following transitions were monitored: *m/z* 580.25 to 173.10 (Q) and 408.20 (q) for β‐CM5, *m/z* 590.40 to 173.10 (Q) and 418.20 (q) for β‐CM5‐d_10_. The ratio of the peak area of β‐CM5 product ion (*m/z* 173.10) to that of the corresponding β‐CM5‐d_10_ product ion was used to quantify β‐CM5. Concentrations were calculated based on the calibration curve and peak area ratios obtained from milk extracts. Each calibration solution (0, 1, 5, 10, 50, and 100 ng/mL) also containing β‐CM5‐d_10_ (500 ng/mL) was prepared in an ultra‐pure water/MeOH mixture (70:30, v/v). β‐CM5 content, expressed in ng/g of milk, in PM samples was calculated based on its content obtained in freeze‐dried milk samples and ratios of liquid milk to freeze‐dried milk powder (Table S1). Meanwhile, β‐CM5 content, expressed in ng/g of milk, in IP samples was calculated based on its content obtained in liquid milk. The method limit of detection (LOD) and the method limit of quantification (LOQ) were determined based on signal‐to‐noise ratios (S/N) of 3 and 10, respectively, at the lowest concentration of β‐CM5 in a milk sample (Nguyen et al. 2014).

## 3 Results and Discussion

### 3.1 LC–MS/MS Analysis for Beta‐Casomorphin 5

LC–MS/MS is a sensitive analytical method widely used to determine peptide content in food, particularly β‐CM7 in dairy products and in in vitro gastrointestinal digests of dairy products (Juan‐García et al. 2009, De Noni and Cattaneo 2010; Nguyen et al. 2015, 2019, 2021; Asledottir et al. 2017; Lambers et al. 2021). To date, however, β‐CM5 has not been detected in pasteurized, sterilized, UHT and in‐bottle sterilized milk (Juan‐García et al. 2009; De Noni and Cattaneo 2010; Nguyen et al. 2015) or in in vitro gastrointestinal digests of these dairy products (De Noni and Cattaneo 2010; Nguyen et al. 2021; Lambers et al. 2021). β‐CM5 content appears to occur in raw milk at a very low level of 0.64 ng/g of milk (Nguyen et al. 2015), and therefore, the detection of β‐CM5 is strongly dependent on the sensitivity and selectivity of the analytical method. Consequently, β‐CM5 levels below the detection limit of published methods may have remained undetected. In this study, an LC–MS/MS system (Triple quadrupole mass spectrometer LCMS‐8045 Shimadzu, Japan) and an MRM approach were developed to identify and quantify β‐CM5 in PM. In addition, Strata‐X‐PRO cartridges with a new polymeric sorbent (Phenomenex, NSW, Australia) were used to purify milk extracts before LC–MS/MS analysis, improving the method's sensitivity.

To identify β‐CM5 using LC–MS/MS techniques, a minimal four‐point identification approach was used. Additionally, two MRM transitions were monitored, along with retention time (RT), to identify this opioid pentapeptide in sample extracts. The retention time deviation was set to RT ≤ 1%, and the absolute difference in RT for β‐CM5 between the sample and reference did not exceed the limit of 0.1 min (Table 1). In addition, the ion ratios for MRM transitions were calculated and reviewed to confirm β‐CM5 in milk extracts according to 2002/657/EC regulations (European Commission 2021).

**TABLE 1:** MRM ratio and retention time for LC–MS/MS performance.

Samples	MRM ratio^a^ (%, mean ± SD, *n* = 2)	Permitted tolerance	RT (min, mean ± SD, *n* = 2)	Deviation of RT^b^ (%)
Standard solutions	42.40 ± 3.30	31.80–53.00	3.26 ± 0.00	—
PM1	41.11 ± 4.50	—	3.27 ± 0.01	0.15
PM2	35.01 ± 5.71	—	3.27 ± 0.00	0.18
PM3	45.49 ± 6.03	—	3.28 ± 0.01	0.20
PM4	41.43 ± 6.06	—	3.26 ± 0.02	0.30
PM5	40.29 ± 10.43	—	3.25 ± 0.01	0.12

*Note:* PM1, 2, 3, 4, and 5: full‐fat PM 1, 2, 3, 4 and 5.

Abbreviations: RT: retention time; SD: standard deviation.

^a^ MRM ratios (multiple reaction monitoring) were 408.2/173.1.

^b^ RT ratios of β‐CM5 to β‐CM5‐d10 in all milk samples corresponded to that of standard solutions.

Table 1 shows that MRM ratios obtained from all milk samples ranged from 35.01 to 45.49, being within the permitted tolerance (31.80–53.00). Furthermore, the absolute differences in RT between the milk samples and the internal standard ranged from 0.00 to 0.02 min and RT deviations were between 0.12 and 0.30. Therefore, these results unequivocally demonstrate the presence of β‐CM5 in PM.

Results showed that the developed LC–MS/MS analytical technique had good linearity with R^2^ of 0.9997. The developed method also had a high sensitivity, which was demonstrated by the method limit of detection (LOD) and the method limit of quantification (LOQ) calculated at signal‐to‐noise (S/N) ratios of 3 and 10, respectively. LOD and LOQ were 0.03 and 0.11 ng/g, respectively. These results were lower than those observed in the previous study (Nguyen et al. 2015).

### 3.2 Beta‐Casomorphin 5 in Pasteurized Milk

Previous studies reported that β‐CM5 was not detected in PM samples (De Noni and Cattaneo 2010; Nguyen et al. 2015), UHT, and in‐bottle sterilized milk (De Noni and Cattaneo 2010). In contrast, LC–MS/MS analysis of five commercial PM samples (PM1–PM5) from different manufacturers in the present study revealed the presence of β‐CM5 in all PM samples (Figure 2). The results showed that β‐CM5 contents varied considerably among PM samples, with the lowest content observed in the PM5 sample (0.10 ng/g < LOQ) and the highest content obtained from the PM1 sample (0.55 ng/g) (Table 2). The findings suggest that β‐CM5 was potentially carried over from raw milk, has been released by endogenous enzymes, or formed by bacterial exogenous enzymes during product storage or heat treatment. Biologically, these results indicate that the levels of β‐CM5 in PM (0.12–0.55 ng/g, corresponding to approximately 0.21.10^−3^–0.95.10^−3^ μmol/L) remain below the IC_50_ thresholds reported in bioassay studies (Brantl et al. 1981; Koch et al. 1985). Moreover, previous studies have demonstrated that β‐CM5 is not generated during in vitro gastrointestinal digestion (De Noni and Cattaneo 2010; Nguyen et al. 2021). Overall, these low levels indicate that daily consumption of PM is unlikely to trigger opioid activity.

**FIGURE 2:**
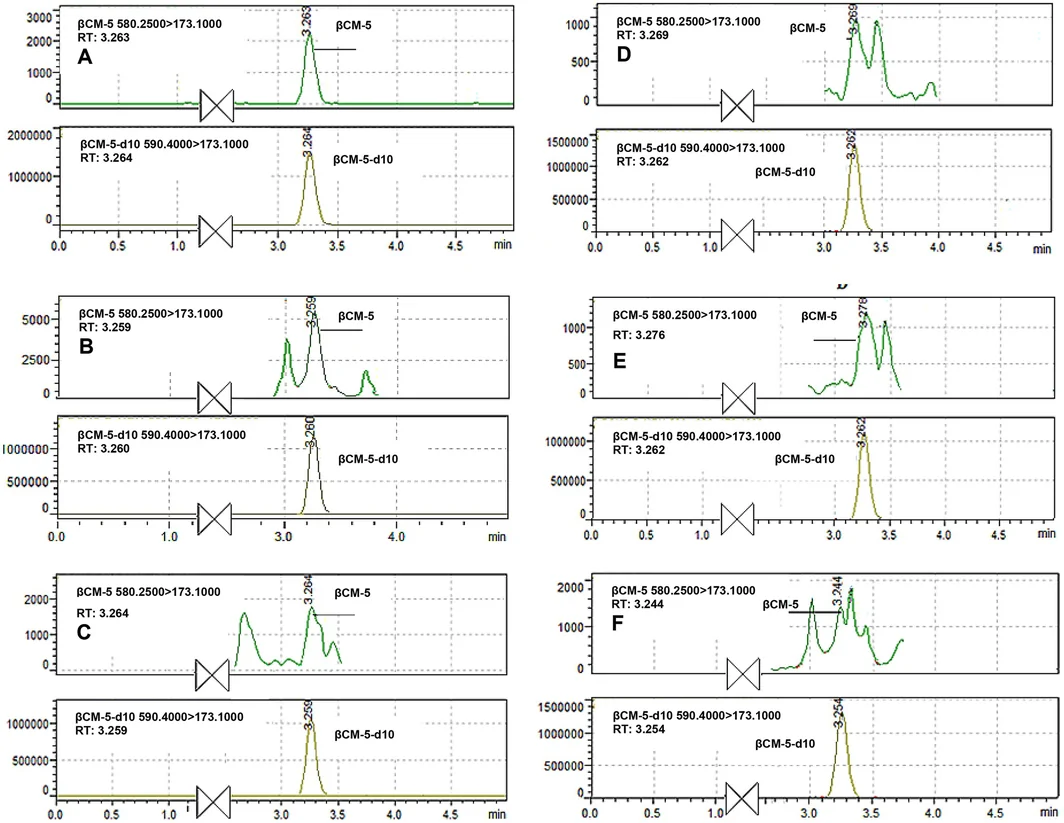
LC–MS/MS chromatogram of β‐casomorphin 5 and β‐casomorphin 5‐d10 in a standard solution (A); and PM1 (B), PM2 (C), PM3 (D), PM4 (E), and PM5 (F). RT, retention time.

**TABLE 2:** The level of β‐casomorphin 5 in pasteurized milk.

Milk samples	β‐casomorphin 5 (ng/g, mean ± SD, *n* = 2)
PM1	0.55 ± 0.05
PM2	0.16 ± 0.02
PM3	0.16 ± 0.01
PM4	0.12 ± 0.01
PM5	< LOQ

*Note:* LOQ: method limit of quantification, 0.11 ng/g. PM1, 2, 3, 4, and 5: full‐fat PM 1, 2, 3, 4 and 5.

Abbreviation: SD: standard deviation.

Pasteurization processing plays a crucial role in dairy manufacture. It is applied to effectively inactivate enzymes and pathogenic microorganisms to extend the shelf life of dairy products. However, some endogenous enzymes, such as plasmin and cathepsin D, and certain bacterial proteases released from *Pseudomonas* species may not be completely inactivated during this heat treatment (Elisa et al. 2026). In addition, this process can also lead to protein modifications (Nguyen et al. 2021). In the present study, IP samples supplied by a dairy processor were used to evaluate the effect of industrial heat treatment on β‐CM5. The content of β‐CM5 in IP samples is shown in Table 3. The results revealed that β‐CM5 content remained relatively stable after heat treatment of milk, which were 0.11 ng/g in raw milk, and 0.13 ng/g and 0.10 ng/g for heated milk samples at 62°C/5 min and 72°C/5 min, respectively (Table 3).

**TABLE 3:** The effect of heat treatment on β‐camosorphin 5.

Milk samples	The level of β‐camorsophin 5^a^ (ng/g milk)
Raw milk	0.11 ± 0.00
62°C/5 min	0.13 ± 0.04
72°C/5 min	0.10 (<LOQ)

*Note:* LOQ: Limit of quantitation, 0.11 ng/g.

^a^ Mean value ± SD (standard deviation, *n* = 2).

In theory, β‐CM5, which corresponds to fragment f60‐64 of β‐caseins, can be released from β‐caseins by enzymes or heat treatment. It is worth noting that the raw milk used in the present study was collected from a healthy herd and then immediately subjected to heat treatment. The raw milk was transported in airtight bags and under cold conditions to the laboratory in a short time. Therefore, the presence of β‐CM5 in the raw milk found in the present study likely resulted from the hydrolysis of β‐caseins by endogenous proteases, such as plasmin and cathepsins, rather than exogenous enzymes derived from bacteria.

It's widely acknowledged that heat treatment has the potential to release peptides due to the impact of radicals generated during the Maillard reaction on the protein backbone (Meltretter et al. 2008; Ebner et al. 2016). However, results found in the present study suggest that industrial pasteurization treatments were unlikely to release β‐CM5. This finding agrees with data reported by Nguyen et al. (2021). Therefore, the variation in β‐CM5 content among the five milk brands is likely attributed to differences in milk source, which may contain different β‐casein content and endogenous enzyme activity.

## 4 Conclusions

The integration of LC–MS/MS with the stable isotope‐labeled internal standard and solid‐phase extraction (SPE) purification provides a highly reliable analytical approach for the quantification of β‐CM5 in pasteurized milk. These combined analytical techniques have strong potential to be used as a “gold standard” method for the determination of β‐CM5 in various dairy products. Overall, this study provides new insights into the occurrence of β‐CM5 in PM and demonstrates that pasteurization treatments were unlikely to release β‐CM5. The variation in β‐CM5 content among PM samples from different milk brands was likely attributable to differences in raw milk sources. Future studies of additional factors, such as raw milk β‐casein profiles and endogenous activity, should be further investigated to fully elucidate the mechanisms governing β‐CM5 content in dairy products.

However, a limitation of the present study is that the absolute extraction recovery of β‐CM5 and its stability during sample lyophilization and storage at −20°C were not independently evaluated. Although an isotope‐labeled internal standard was used to compensate for potential losses during subsequent extraction and LC–MS/MS analysis, potential changes in β‐CM5 occurring before spiking the internal standard cannot be excluded. In addition, each milk brand was evaluated using a single 50g sample, with n=2 representing analytical replicates derived from the same homogenized, freeze‐dried powder rather than independent biological or batch replicates. Consequently, inter‐batch variability within the same brand was not assessed in this study.

## Author Contributions

**Duc Doan Nguyen:** methodology, investigation, writing – original draft, writing – review and editing. **Vicky Solah:** conceptualization, project administration, writing – review and editing. **Suzanne Daubney:** conceptualization, resources, writing – review and editing. **Malgorzata Kowalczyk:** methodology, writing – review and editing. **Wendy Hunt:** writing – review and editing.

## Funding

This work was funded by the AusIndustry Entrepreneurs' Programme, Innovation Connections with Bannister Downs Dairy, Grant number ICG002310.

## Consent

All authors agree to publish this article.

## Conflicts of Interest

The authors declare no conflicts of interest.

## Supporting information


**Table S1:** Ratios of liquid milk to freeze‐dried milk powder.

## Data Availability

The data used to support the findings of this study are included in the article.

## References

[fsn372484-bib-0001] Asledottir, T. , T. T. Le , B. Petrat‐Melin , T. G. Devold , L. B. Larsen , and G. E. Vegarud . 2017. “Identification of Bioactive Peptides and Quantification of β‐Casomorphin‐7 From Bovine β‐Casein A1, A2 and I After Ex Vivo Gastrointestinal Digestion.” International Dairy Journal 71: 98–106. 10.1016/j.idairyj.2017.03.008.

[fsn372484-bib-0002] Brantl, V. , H. Teschemacher , J. Bläsig , A. Henschen , and F. Lottspeich . 1981. “Opioid Activities of β‐Casomorphins.” Life Sciences 28: 1903–1909. 10.1016/0024-3205(81)90297-6. 6265721

[fsn372484-bib-0003] Brantl, V. , H. Teschemacher , A. Henschen , and F. Lottspeich . 1979. “Novel Opioid Peptides Derived From Casein (β‐Casomorphins). I. Isolation From Bovine Casein Peptone.” Hoppe‐Seyler's Zeitschrift für Physiologische Chemie 360: 1211–1216. 10.1515/bchm2.1979.360.2.1211. 511110

[fsn372484-bib-0004] Daniloski, D. , N. A. McCarthy , and T. Vasiljevic . 2021. “Bovine β‐Casomorphins: Friends or Foes? A Comprehensive Assessment of Evidence From in Vitro and Ex Vivo Studies.” Trends in Food Science & Technology 116: 681–700. 10.1016/j.tifs.2021.08.003.

[fsn372484-bib-0005] De Noni, I. , and S. Cattaneo . 2010. “Occurrence of β‐Casomorphins 5 and 7 in Commercial Dairy Products and in Their Digests Following in Vitro Simulated Gastro‐Intestinal Digestion.” Food Chemistry 119: 560–566. 10.1016/j.foodchem.2009.06.058.

[fsn372484-bib-0006] De Noni, I. , M. Stuknyte , and S. Cattaneo . 2015. “Identification of β‐ Casomorphins 3 to 7 in Cheeses and in Their in Vitro Gastrointestinal Digestates.” LWT‐ Food Science and Technology 63: 550–555. 10.1016/j.lwt.2015.03.036.

[fsn372484-bib-0007] Ebner, J. , F. Baum , and M. Pischetsrieder . 2016. “Identification of Sixteen Peptides Reflecting Heat and/or Storage Induced Processes by Profiling of Commercial Milk Samples.” Journal of Proteomics 147: 66–75. 10.1016/j.jprot.2016.03.021. 27016039

[fsn372484-bib-0008] Elisa, G. , Z. Nora , S. Joachim , and D. Sevim . 2026. “Comprehensive Analysis of Peptide Profiles in Pasteurized Milk: Seven Key Peptides as Biomarkers for Storage‐Dependent Proteolysis.” Food Chemistry 499: 147280. 10.1016/j.foodchem.2025.147280. 41330128

[fsn372484-bib-0009] Elliott, R. B. , D. P. Harris , J. P. Hill , N. J. Bibby , and H. E. Wasmuth . 1999. “Type I (Insulin‐Dependent) Diabetes Mellitus and Cow Milk: Casein Variant Consumption.” Diabetologia 42: 292–296. 10.1007/s001250051153. 10096780

[fsn372484-bib-0010] European Commission . 2021. “EU 2021/808 on the Performance of Analytical Methods for Residues of Pharmacologically Active Substances Used in Food‐Producing Animals and on the Interpretation of Results As Well As on the Methods to be Used for Sampling and Repealing Decisions 2002/657/EC and 98/179/EC.”

[fsn372484-bib-0011] Jianqin, S. , X. Leiming , X. Lu , G. W. Yellan , J. Ni , and A. J. Clarke . 2016. “Effects of Milk Containing Only A2 Beta Casein Versus Milk Containing Both A1 and A2 Beta Casein Proteins on Gastrointestinal Physiology, Symptoms of Discomfort, and Cognitive Behavior of People With Self‐Reported Intolerance to Traditional Cows' Milk.” Nutrition Journal 15: e35. 10.1186/s12937-016-0147-z. PMC4818854 27039383

[fsn372484-bib-0012] Juan‐García, A. , G. Font , C. Juan , and Y. Picó . 2009. “Nanoelectrospray With Ion‐Trap Mass Spectrometry for the Determination of Beta‐Casomorphins in Derived Milk Products.” Talanta 80: 294–306. 10.1016/j.talanta.2009.06.062. 19782229

[fsn372484-bib-0013] Koch, G. , K. Wiedemann , and H. Teschemacher . 1985. “Opioid Activities of Human β‐Casomorphins.” Naunyn‐Schmiedeberg's Archives of Pharmacology 331: 351–354. 10.1007/BF00500818. 3005882

[fsn372484-bib-0014] Lambers, T. T. , S. Broeren , J. Heck , M. Bragt , and T. Huppertz . 2021. “Processing Affects Beta‐Casomorphin Peptide Formation During Simulated Gastrointestinal Digestion in Both A1 and A2 Milk.” International Dairy Journal 121: e105099. 10.1016/j.idairyj.2021.105099.

[fsn372484-bib-0015] Laugesen, M. , and R. Elliott . 2003. “Ischaemic Heart Disease, Type 1 Diabetes, and Cow Milk A1 Beta‐Casein.” New Zealand Medical Journal 116: U295. 12601419

[fsn372484-bib-0016] Meltretter, J. , A. Schmidt , A. Humeny , C. M. Becker , and M. Pischetsrieder . 2008. “Analysis of the Peptide Profile of Milk and Its Changes During Thermal Treatment and Storage.” Journal of Agricultural and Food Chemistry 56: 2899–2906. 10.1021/jf073479o. 18419126

[fsn372484-bib-0017] Nguyen, D. D. , F. Busetti , S. K. Johnson , and V. A. Solah . 2015. “Identification and Quantification of Native Beta‐Casomorphins in Australian Milk by LC‐MS/MS and LC‐HRMS.” Journal of Food Composition and Analysis 44: 102–110. 10.1016/j.jfca.2015.08.009.

[fsn372484-bib-0018] Nguyen, D. D. , F. Busetti , G. Smolenski , S. K. Johnson , and V. A. Solah . 2021. “Release of Beta‐Casomorphins During In‐Vitro Gastrointestinal Digestion of Reconstituted Milk After Heat Treatment.” LWT‐ Food Science and Technology 136: e110312. 10.1016/j.lwt.2020.110312.

[fsn372484-bib-0019] Nguyen, D. D. , V. A. Solah , S. K. Johnson , J. W. A. Charrois , and F. Busetti . 2014. “Isotope Dilution Liquid Chromatography–Tandem Mass Spectrometry for Simultaneous Identification and Quantification of Beta‐Casomorphin 5 and Beta‐Casomorphin 7 in Yoghurt.” Food Chemistry 146: 345–352. 10.1016/j.foodchem.2013.09.057. 24176353

[fsn372484-bib-0020] Nguyen, D. D. , V. A. Solah , S. K. Johnson , et al. 2019. “Identification and Quantification of Beta‐Casomorphin Peptides Naturally Yielded in Raw Milk by Liquid Chromatography‐Tandem Mass Spectrometry.” LWT‐Food Science and Technology 111: 465–469. 10.1016/j.lwt.2019.05.074.

[fsn372484-bib-0021] Pal, S. , K. Woodford , S. Kukuljan , and S. Ho . 2015. “Milk Intolerance, Beta‐Casein and Lactose.” Nutrients 7: 7285–7297. 10.3390/nu7095339. PMC4586534 26404362

[fsn372484-bib-0022] Shivam, K. D. , T. Abhishek , K. J. Manoj , et al. 2024. “Effect of Bovine Beta‐Casomorphins on Rat Pancreatic Beta Cells (RIN‐5F) Under Glucotoxic Stress.” Biochemical and Biophysical Research Communications 739: 150578. 10.1016/j.bbrc.2024.150578. 39178795

[fsn372484-bib-0023] Sienkiewicz‐Szłapka, E. , B. Jarmołowska , S. Krawczuk , E. Kostyra , H. Kostyra , and M. Iwan . 2009. “Contents of Agonistic and Antagonistic Opioid Peptides in Different Cheese Varieties.” International Dairy Journal 19: 258–263. 10.1016/j.idairyj.2008.10.011.

[fsn372484-bib-0024] Simonetta, C. , P. Gianluca , D. P. Sabrina , et al. 2025. “Impact of β‐Casein Variants on the Formation of β‐Casomorphins in Blue Cheeses: Investigating Key Drivers Through Peptidomic Analysis.” Food Research International 201: 115556. 10.1016/j.foodres.2024.115556. 39849707

[fsn372484-bib-0025] Sokolov, O. , N. Kost , O. Andreeva , et al. 2014. “Autistic Children Display Elevated Urine Levels of Bovine Casomorphin‐7 Immunoreactivity.” Peptides 56: 68–71. 10.1016/j.peptides.2014.03.007. 24657283

[fsn372484-bib-0026] Thiruvengadam, M. , B. Venkidasamy , P. Thirupathi , I. M. Chung , and U. Subramanian . 2021. “β‐Casomorphin: A Complete Health Perspective.” Food Chemistry 337: 127765. 10.1016/j.foodchem.2020.127765. 32799161

[fsn372484-bib-0027] Wasilewska, J. , M. Kaczmarski , E. Kostyra , and M. Iwan . 2011. “Cow's‐Milk–Induced Infant Apnoea With Increased Serum Content of Bovine β‐Casomorphin‐5.” Journal of Pediatric Gastroenterology and Nutrition 52: 772–775. 10.1097/MPG.0b013e318204bbcc. 21478761

