# Supplementary material for: A Sensitive LC–MS/MS Method for Quantifying Beta‐Casomorphin 5 in Pasteurized Milk
Source: Food Sci Nutr. 2026 Oct 19;14(10):e72484. doi: 10.1002/fsn3.72484 (PMC13680660; doi:10.1002/fsn3.72484)
Supplement: Supplementary file 1 [file FSN3-14-e72484-s001.docx]

Table S1: Ratios of liquid milk to freeze-dried milk powder

| Milk | Liquid milk (g) | Freeze-dried milk powder (g) | Liquid milk/freeze-dried milk powder ratios | Sample of freeze-dried milk powder (g) | Sample of liquid milk (converted) (g) |
| --- | --- | --- | --- | --- | --- |
| PM1 | 50.00 | 9.14 | 5.47 | 1.0732 | 5.8704 |
| PM1 | 50.00 | 9.14 | 5.47 | 1.0134 | 5.5433 |
| PM2 | 49.99 | 6.32 | 7.91 | 1.0164 | 8.0397 |
| PM2 | 49.99 | 6.32 | 7.91 | 1.0756 | 8.5080 |
| PM3 | 50.00 | 12.14 | 4.12 | 1.1505 | 4.7401 |
| PM3 | 50.00 | 12.14 | 4.12 | 1.1329 | 4.6675 |
| PM4 | 50.00 | 6.80 | 7.35 | 1.0281 | 7.5565 |
| PM4 | 50.00 | 6.80 | 7.35 | 1.0607 | 7.7961 |
| PM5 | 50.00 | 6.60 | 7.58 | 1.0037 | 7.6080 |
| PM5 | 50.00 | 6.60 | 7.58 | 1.0132 | 7.6801 |
